# Moderation between resting-state connectivity and brain amyloid levels on speed of cognitive and physical function in older adults: Evidence for network-based cognitive reserve

**DOI:** 10.52294/001c.141046

**Published:** 2025-07-22

**Authors:** Paul J. Laurienti, Stephen B. Kritchevsky, Robert G. Lyday, Chal E. Tomlinson, Michael E. Miller, Samuel N. Lockhart, Melissa M. Rundle, Christina E. Hugenschmidt, Jonathan H. Burdette, Heather M. Shappell, Haiying Chen, Laura D. Baker, Blake R. Neyland, Roee Holtzer

**Affiliations:** 1 Department of Radiology, Wake Forest University School of Medicine; 2 Sticht Center for Healthy Aging and Alzheimer’s Prevention, Department of Internal Medicine Section on Gerontology and Geriatric Medicine, Wake Forest University School of Medicine; 3 Johnson & Johnson (United States); 4 Division of Public Health Sciences, Wake Forest University School of Medicine; 5 Department of Biostatistics and Data Science, Wake Forest University School of Medicine; 6 LifeSci Consulting; 7 Department of Neurology, Albert Einstein College of Medicine

**Keywords:** amyloid, network, aging, moderation, cognitive reserve

## Abstract

Cognitive and physical function are interrelated in aging. Co-occurring impairments in both domains can be debilitating and lead to increased risk of developing dementia. Amyloid beta (Aβ) deposition in the brain is linked to cognitive decline and is also associated with poorer physical function in older adults. However, significant inter-individual variability exists with respect to the influence of increased brain Aβ concentrations on cognitive and physical outcomes. Identifying factors that explain inter-individual variability in associations between Aβ and clinical outcomes could inform interventions designed to delay declines in both cognitive and physical function. Cognitive reserve (CR) is considered a buffer that allows for cognitive performance that is better than expected for a given level of brain injury or pathology. Although the neural mechanisms underlying CR remain unknown, there is growing evidence that resting-state brain networks may serve as a neural surrogate for CR. The current study utilized a statistical interaction model to evaluate if functional brain networks moderated associations between brain Aβ and cognitive and physical function in community-dwelling older adults from the Brain Networks and Mobility (B-NET) study. Significant moderations were found between Aβ levels and both the central executive and subcortical networks with cognitive and physical function. The findings suggest that brain networks serve as a buffer against the influence of Aβ accumulation on cognitive and physical function indicating that the network integrity could one of the neural mechanisms supporting CR.

## INTRODUCTION

Among older adults, common and debilitating impairments in cognitive^1,2^ and physical^3^ function result in adverse health outcomes and increased economic burden.^4–7^ Furthermore, cognitive and physical function are interrelated in aging,^8–10^ and co-occurring impairments in both domains lead to increased risk of developing dementia.^11^ Amyloid beta (Aβ) is an established biomarker of Alzheimer’s disease^12,13^ and has been linked to cognitive decline among older adults.^14^ Aβ is also associated with poorer physical function in older adults.^15,16^ However, significant inter-individual variability exists with respect to the influence of increased brain Aβ concentrations on cognitive and motoric outcomes. Although the reasons for this variability remain largely unknown, factors such as education, other brain pathology, and cognitive impairment influence the effect of amyloid on cognitive or physical function.^17,18^ For example, those individuals with impairment are more likely to exhibit associations between amyloid burden and poor physical function.^19–22^ Hence, identifying common modifiable protective factors that could attenuate age- and disease-related declines in cognitive and physical function is paramount. Specifically, identifying factors that explain inter-individual variability in associations between Aβ and clinical outcomes among older adults without dementia could inform interventions designed to delay declines in both cognitive and physical domains of function.

Cognitive reserve (CR) is considered a buffer that confers individual differences in adapting to and executing tasks in the context of adverse effects of aging, disease, or injury to brain structure and function.^23^ Considerable work has been dedicated to clarifying definitions and converging on common language to discuss CR, including a Resilience and Reserve Collaboratory that serves as a great resource (https://reserveandresilience.com/). Extensive research,^17,18^ including a recent meta-analysis,^24^ demonstrates robust protective effects for CR against cognitive decline and dementia. In contrast, literature concerning the role of CR in mitigating age-related effects on physical outcomes is scarce. Recent studies, however, demonstrated that, among community-residing older adults, higher CR is associated with more efficient brain activation patterns implicated in single and dual-task walking,^25^ and lower odds of developing incident mobility impairment.^26,27^ Combined, these findings suggest a more generalized role for CR in mitigating risk of developing adverse health outcomes wherein individuals with higher CR levels are less susceptible to both cognitive and motoric declines.

The assessment of CR, however, is controversial as various proxy measures and methods have been utilized to operationalize this hypothetical construct.^18^ One approach is to identify measures of brain network connectivity that correlate with CR or modify the associations between neuropathological measures and clinical outcomes. Using functional magnetic resonance imaging (fMRI) to assess resting-state blood oxygenation level dependent (BOLD)^28^ to characterize brain network properties has the advantage that the measures are task-invariant and have been shown to be replicable across studies, populations, and neuroimaging modalities.^29^ Indeed, increased resting-state connectivity, notably in frontal regions, is associated with CR, and modifies the associations between cortical thickness and cognitive function.^30^ Converging evidence further suggests that increased functional connectivity at rest in executive frontal networks may be considered a functional neural marker of CR.^31–33^ In addition, increased functional organization of brain networks is associated with higher socio-demographic and performance measures of CR,^34^ and attenuates the negative effect of neuropathological markers of Alzheimer’s disease on cognitive decline.^35^ The strength of functional network connectivity in an episodic memory network mediated the relationship between brain tau levels measured using positron emission tomography (PET) and episodic memory above what was accounted for by cortical thickness in cognitively unimpaired adults.^36^ This finding does not imply CR *per se* but does indicate that the network connectivity is the neural mechanism linking pathology measures to cognition. In sum, there is conceptual and empirical support to using resting-state brain networks as a neural surrogate for CR. However, whether inter-individual differences in resting-state network measures of CR explain variability in associations between Aβ and cognitive and motor outcomes in healthy older adults has not been reported.

The current study addressed this critical gap in the literature. Specifically, we evaluated if there was a significant moderating effect between functional brain network integrity and brain Aβ (measured using PET) in associations with baseline and follow-up measures of cognitive and physical function. We used data from the Brain Networks and Mobility (B-NET) study,^37^ a longitudinal study, to determine whether brain network community structure^38^ could shed light on cortical involvement in cognitive and physical function and decline among older adults. We employed an unbiased empirical approach to operationalize neural representation of CR using brain network community structure^38^ in eight canonical resting-state networks commonly identified across different methods.^39–46^ In each participant, network nodes were assigned into a common community if they were more connected with each other than with other network communities. The spatial alignment of each participant’s community structure with the eight intrinsic networks^47^ was quantified as a measure of network integrity. We hypothesized that there would be a statistical interaction (moderation) between spatial integrity of the network community structure and Aβ levels in association with cognitive and physical function. Specifically, we predicted that higher CR (greater intrinsic network integrity in the current study) would attenuate negative associations between Aβ concentrations and speed of cognitive and physical function. The digit symbol substitution test (DSST)^48^ was used as a measure of cognitive processing speed. The expanded short physical performance battery (eSPPB),^49^ a composite physical measure that includes multiple timed assessments, was used to assess physical function. These ecologically valid measures were selected because they are simple enough to administer in clinical situations, are associated with long-term clinical outcomes, including disability and mortality,^50–55^ and potentially share common neural mechanisms.^56^

## METHODS

### PARTICIPANTS

B-NET was a longitudinal, observational trial of community-dwelling older adults aged 70 and older recruited from Forsyth County, NC, that enrolled 192 participants. Participants were asked to complete a baseline visit along with follow-up visits at 6, 18, and 30 months. Brain MRIs that were collected at baseline and cognitive and physical function scores from baseline and 30 months were used in the current analyses. The current study focuses on 81 participants that participated in an ancillary study that included positron emission tomography (PET) imaging. All participants gave written informed consent in this study as approved by the Wake Forest University School of Medicine Institutional Review Board (IRB, protocol #IRB00046460).

The following were exclusion criteria for the parent study: hospitalization or surgery within the past 6 months, uncontrolled or serious chronic disease, uncorrected major hearing or vision problems, single or double amputee, musculoskeletal implants that limit physical functional testing (e.g., joint replacements), dependency on assistance for ambulation, clinical diagnosis of any disease affecting mobility (e.g., Parkinson’s disease), psychotic disorder, alcohol use disorder, history of traumatic brain injury or brain tumor, recent history of seizures, or a score of 20 or lower on the Montreal Cognitive Assessment (MoCA). MOCA scores from 21–25, along with other cognitive test results, were reviewed by the study neuropsychologist to determine eligibility on an individual basis. Other exclusion criteria included the inability/unwillingness to complete a brain MRI or PET scan, plans to move from the area within 24 months, or current participation in a behavioral intervention trial.

### COGNITIVE AND PHYSICAL FUNCTION ASSESSMENTS

The Digit-Symbol Substitution Test (DSST)^48^ was used to assess cognitive processing speed. This is a paper and pencil test where digits 1–9 are associated with a symbol. The digit-symbol assignments are displayed at the top of the page. Below that are rows of digits with empty boxes below each digit. The participants are instructed to copy the symbol associated with each digit. They are first given 7 practice items and then instructed to fill in as many of the remaining boxes as they can in two minutes. The total number of correct symbols is the final score that was used in the analyses.

The Expanded Short Physical Performance Battery (eSPPB)^49^ was used to assess overall physical function. This test was adapted from the original SPPB^53^ to be used in well-functioning populations. The four components of the eSPPB include balance, four-meter walk, narrow four-meter walk, and a chair stand. The balance assessment includes a side-by-side posture, semi-tandem, tandem, and one-leg positions. For the 4-meter walk participants are timed walking at their usual speed for two trials and the fastest is used. For the “narrow walk” participants are given 3 attempts to complete two successful walks not stepping outside of a 20 cm path. For the chair stand, participants were timed while standing up from a seated position 5 times without using their arms. Scores for each test within the eSPPB assessment ranged from 0–1 based on a ratio of the measured value to the best possible performance. Adding across the four components gives a continuous score from 0–4.

### A*β* PET

PET scans began approximately one year after the parent study, with the average time between the participant’s baseline visit and the subsequent PET scan being 260.64 days. PET scans were not completed at any of the follow-up visits. [11C]PiB was used to assess fibrillar Aβ brain deposition on PET. Following a CT scan for attenuation correction, participants were injected with ~370 MBq [11C]PiB and scanned on a 64-slice GE Discovery MI DR PET/CT.^57^ Centiloid (CL) analysis^58^ was conducted in PMOD v4.1 (PMOD LLC Technologies, Switzerland). PET frames were aligned to a 3D T1-weighted MRI, and a static PET image was created by averaging 50–70 min (5-min frames) post-injection data. The MRI and aligned average PET scan were input into the PMOD PNEURO Step-wise Maximum Probability Atlas workflow using the CL atlas template. MRIs were normalized to MNI-space template and segmented, with coregistered PET scans normalized to MNI space using MRI parameters. SUVr was calculated using the standard MNI-space CL ROI (whole cerebellum reference), and CL scores were calculated using Klunk et al.^58^ equation 1.3b (CL =100(SUVr-1.009)/1.067). This method was validated using the GAAIN data set. All reports of Aβ levels are the whole brain quantitative CL scores. All analyses used CL scores as a continuous measure, but for descriptive purposes, a cut-off of >24 was used to identify positive scans.^59^

### MRI COLLECTION, PROCESSING, AND NETWORK GENERATION

#### IMAGE ACQUISITION

All participants completed brain MRI scans at baseline and at the 30-month follow-up. The current study used the baseline MRI scans for all analyses. Scans included a T1-weighted 3D volumetric MPRAGE anatomical image (TR=2300ms; TE=2.98ms; number of slices=192; voxel dimensions = 1.0×1.0×1.0mm; FOV=256mm; scan duration=312s), T2 FLAIR images (TR=4800ms; TE=4.41ms; number of slices=160; voxel dimensions=1.0×1.0×1.2mm; FOV=256mm; scan duration=293s), and resting-state blood oxygenation level-dependent (BOLD) images (TR=2000ms; TE=25ms; number of slices=35; voxel dimensions=4.0×4.0×5.0mm; FOV=256mm; scan duration=7m 20s). During the resting-state fMRI scan, a fixation cross was displayed on the monitor. Other image sequences were performed but are not used in the current study.

#### STRUCTURAL IMAGE PROCESSING

Images were preprocessed using Statistical Parametric Mapping version 12 (SPM12, http://www.fil.ion.ucl.ac.uk/spm), and Advanced Normalization Tools (ANTs). The T1 weighted images were segmented into grey matter (GM) and white matter (WM) and cerebrospinal fluid (CSF) using SPM-12. The resulting GM and WM segments were used to calculate GM volume and WM volume, respectively. Intracranial Volume (ICV) was calculated by combining all three (GM, WM, and CSF) segments. Gray and white matter segmented images were summed to generate a mask of brain parenchyma. The summed images were manually cleaned to remove extra-parenchyma tissues using MRI-cron software.^60^ Images were then masked and spatially normalized according to the Montreal Neurological Institute (MNI) template using ANTs.

White matter lesions were calculated using the Lesion Segmentation Toolbox with the lesion prediction algorithm (LPA) with default settings within SPM12. No threshold was applied to the segmented images, resulting in each voxel containing a value that represents the percentage of the voxel that is classified as a lesion. The resultant image was used to calculate white matter lesion volume (WMLV) as a percentage of voxel volume. Volumetric measures based on segmented images (GMV, WMV, WMLV, ICV) are in cubic centimeters and were calculated by summing all voxels’ values from the appropriate image. For example, for GMV, the gray matter volume image was used with each value indicating the volume of gray matter in that voxel. Images were then multiplied by the cubic volume of the voxel and divided by 10^3, resulting in all measures being in cubic centimeters (CC). To calculate the volume of structural brain regions (hippocampus, thalamus, prefrontal cortex), the appropriate brain atlases were warped to each subject’s native space to calculate accurate volumes. The volume of each structure was determined in cubic centimeters. The Automated Anatomical Labelling Atlas 2 (AAL2)^61^ was used to calculate volume of hippocampus (regions 41 and 42) and thalamus (regions 81 and 82) volumes. The prefrontal cortex volume was calculated with regions 5, 6 and 7 from the Sallet Dorsal Frontal Parcellation.^62^

#### FUNCTIONAL IMAGE PROCESSING

The functional images were processed with SPM, ANTs, and FMRIB’s “topup” Software Library (FMRIB Software Library v6.0). Image preprocessing included dropping the first 10 image volumes, fieldmap distortion correction using topup, slice time correction, realignment, coregistration with native-space anatomical images, and warping to MNI space using transformation information from ANTs. Signals from total white matter, total gray matter, total CSF, and the 6 rigid-body motion parameters were regressed from the functional images, and the data were band pass filtered (0.009–0.08Hz). The motion scrubbing procedure developed by Power and colleagues^63^ was applied to the preprocessed images and used to correct residual signal artifacts associated with head motion. The number of images removed in the motion scrubbing procedures was included as a covariate in the statistical analyses to control for head motion.

#### BRAIN NETWORK ANALYSES

##### NETWORK GENERATION

A voxel-wise cross-correlation was performed on time series from each voxel pair. This resulted in a fully-connected, weighted brain network, where each voxel is a node and correlation coefficients between nodes are edges. An empirically determined threshold was calculated to satisfy the equation S=log(N)/log(K), where N is the number of network nodes (~20,000), K= average number of connections per node, and S was set to 2.5 based on prior research.^64^ Correlation coefficients at or above the threshold were set to 1 indicating the presence of a connection, and those below the threshold were set to 0. The threshold was applied to the matrix to dichotomize the data and create a final N × N binary adjacency matrix, Aij. This procedure ensures networks with comparable density across participants.

##### COMMUNITY STRUCTURE

Modularity (Q)^38^ was used to identify network community partitions for each study participant using a dynamic Markov process.^65^ The partitioning procedure was applied to the voxel-wise networks, resulting in each individual participant’s brain network being divided into categorical communities. The community can be mapped back into the brain at the voxel level. Given the exploratory nature of the study, a data-driven approach was used to examine potential community structure relationships across the entire brain. Templates for eight resting-state networks that cover the entire brain and are commonly identified across different imaging methods^39–46^ were used. The subnetworks included the central executive network (CEN) often also referred to as the control network or the frontoparietal network, subcortical network (SCN) that includes basal ganglia, thalamus and limbic regions, default mode network (DMN), sensorimotor network (SMN), dorsal attention network (DAN), salience network (SN), frontotemporal network (FTN) that includes orbital frontal cortex and temporal poles, and visual network (VN).

##### SCALED INCLUSIVITY (SI)

The communities of a participant are not inherently assigned to one of the 8 intrinsic subnetworks. Our goal was to assess the integrity of the subnetworks by determining if the nodes in each subnetwork belonged to coherent communities. For example, the nodes that make up the CEN could belong to a single spatially aligned community, or they could be fragmented into separate communities or even members of communities that more closely align with other subnetworks. To assess the spatial integrity of the subnetworks, the community structure of each participant was compared to the *a priori* subnetwork templates using a quantitative metric called Scaled Inclusivity (SI).^66^ This analysis generates voxel-wise maps for each subnetwork with the voxel values quantifying the alignment of the community containing that voxel with the subnetwork of interest. Every voxel in each community will have the same SI value. The calculation of SI is further detailed in Supplemental Figure 1. Values of SI range between 0 and 1; a hypothetical situation with perfect spatial alignment of the *a priori* subnetwork with a participant’s network community results in a value of 1 for all voxels in that community. In practice each node is assigned a values less than 1 depending on the spatial variability of the individuals communities.^47^ High average SI values in a specific population of people indicate that a particular network community is stable and occupies comparable or consistent brain regions across people. Low SI values indicate that the community is more dispersed with greater variability across people. Individual participant SI maps for each subnetwork were used as a measure of subnetwork spatial integrity in the regression analyses detailed below.

### STATISTICAL ANALYSES

#### COHORT CHARACTERISTICS

Baseline descriptive statistics (i.e., mean, standard deviation, proportions) were calculated separately for the participants in the PET cohort and the remainder of the BNET participants. T-tests were used to compare means between groups for continuous variables, and chi-square tests were used for proportions. Pearson’s correlations were used to assess relationships between brain amyloid and cognitive/physical function. All data plots used in figures were generated with Prism (https://www.graphpad.com). All baseline data are represented with filled circles and 30-month data with filled triangles.

#### STATISTICAL MODERATION (INTERACTION) ANALYSES

The primary objective of this work was to test for moderations between network integrity and Aβ levels on eSPPB and DSST as an assessment of CR. To determine if there is a statistical moderating effect between the variables, a statistical interaction was evaluated using a linear regression model^67,68^ shown below:

(1) $$y=\beta_{1}X_{1}+\beta_{2}X_{2}+\beta_{3}X_{1}*X_{2}+\varepsilon$$

where y is the dependent variable (eSPPB or DSST score depending on the model), $X_{1}$ is the SI map for the network of interest (Net), $X_{2}$ is the amyloid level in centiloids units (Amy), and ε is the residual error. The outcome of interest for this regression model is the interaction term (βNet∗Amy). Other X variables, such as age and education, were included in secondary analyses as covariates.

The independent variable ($X_{1}$ or NET) is actually a spatial map, not a single value as typically used in a regression analysis. It is true that each voxel has a value, but generating some average value across the brain to produce a single variable eliminates the spatial information contained in the map. In addition, running a more traditional voxel-wise regression such as those used for activation studies is not trivial because this would require ~20,000 separate regression models, one for each voxel, because the maps are the independent variable (necessary for testing the interaction model), not the dependent variable as used for activation studies. Furthermore, there is no statistical work demonstrating the validity of a mass univariate analysis with unique models for each voxel, which would be beyond the scope of the current study.

**Figure 1.**
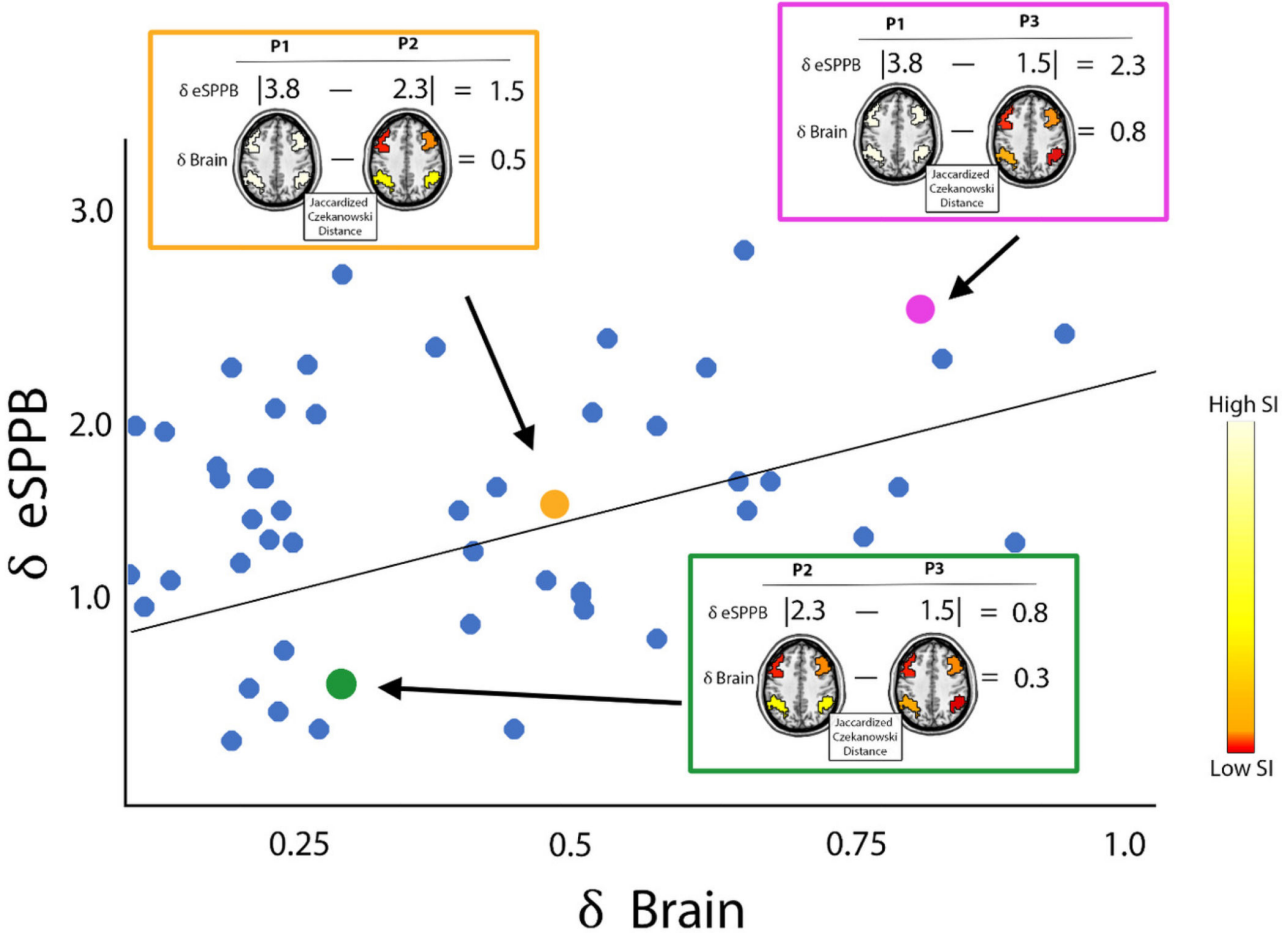
Cartoon depicting the concept behind the distance regression showing how it can capture relationships between the spatial integrity of brain subnetworks assessed with SI and continuous variables such as eSPPB. In this example, the distance between the SI brain images for a subnetwork of interest (∂ Brain) is the independent variable and the distance between the eSPPB scores (∂ eSPPB) is the dependent variable. Distances between each participant pair $\left(ij\right)$ are represented by a point on the scatter plot. The hypothesis is that individuals with similar spatial patterns in their brain maps with have similar behavioral measures. In this cartoon, the hypothetical points for 3 participant (P) pairs are highlighted. Note that the three hypothetical participants shown here are the same participants shown in Figure S1 which demonstrates how SI is calculated to generate voxel-wise maps. Participants 2 and 3 are the pair with the smallest distance between their brain maps, and that is associated with the smallest difference in their eSPPB scores. The Jaccardized Czekanowski Distance is used to compare brain maps and an absolute distance is used for non-image variables. Given that the distances are unsigned, a significant association in the distance regression must be followed up with post-hoc evaluation of the data to determine the direction of the association. In the current cartoon, individuals with high SI values also have high eSPPB scores. Participant 1 has SI values of 1 across the subnetwork and the highest eSPPB while participant 3 has the lowest overall SI and the lowest eSPPB. However, this need not be true as the eSPPB scores could be flipped, but the same distance regions results would be observed. For simplicity, we do not demonstrate a hypothetical interaction between brain network and amyloid as this would require a 3-dimentional cartoon, but the same concepts apply. Also note that the full distance regression is not a simple correlation as shown here, given that every participant is compared to every other participant, necessitating accounting for individual effects.

However, we have recently developed and validated a statistical model that allows the use of a spatial map as an independent variable using a distance regression.^69^ This regression method tests if there is a significant association between the spatial organization of network community structure (the SI maps) and outcomes of interest (eSPPB and DSST in the current study). To achieve this, distances are calculated between each of the variables for every pair of participants to create a distance matrix for each model variable. This converts the spatial maps to distances between participant pairs so that two participants with similar spatial patterns have low distances but two participants with distinct spatial maps have large distances. The premise of the distance regression is that people with similar spatial brain maps (low distances) will also have similar dependent variables with low distances, and vice versa. Figure 1 is a conceptual cartoon depicting how the distance regression identifies associations between the spatial pattern of brain network SI maps and the outcome variables.

Absolute distance was used for all continuous and categorical variables, including the outcome variables. The SI maps were compared using the Jaccardized Czekanowski similarity index^70^ also known as the Ružička index.^71^ Note that this is a similarity index rather than a distance. To be consistent with the distances used for the independent variables, the Jaccard distance (1-Jaccard index) was used for all analyses. A linear model was used to regress physical and cognitive measure distances against predictor variable distances and their interaction. Thus, the final statistical model was more complex than eq. 1 as the actual independent and dependent variables were distances between subject pairs. Conceptually the analysis used the model below, but see^69^ for full details of the distance regression model.

(2) $$\partial y_{ij}=\beta_{1}\partial Net_{ij}+\beta_{2}\partial Amy_{ij}+\beta_{3}\partial Net_{ij}*\partial Amy_{ij}+\varepsilon_{ij}$$

Where $\partial y_{ij}$ is the absolute distance of the outcome variable (eSPPB or DSST) between a pair of participants $\left(ij\right)$. The distances between the independent variable are used for the same subject pairs with the outcome of interest being the interaction between $\partial Net_{ij}$ and $\partial Amy_{ij}$.

Estimation and inference for the regression model were performed using an F test with individual-level effects (ILE) utilizing the distance matrices generated for all ij participant pairs. In our prior work,^69^ we adapted several standard methods for estimation and inference within our distance regression framework: standard F test, F test with individual level effects (ILE), feasible generalized least squares (FGLS), and permutation. We tested these methods across a variety of distance metrics and simulation scenarios. Amongst these, we recommended the F test with ILE as it was among the best at controlling type I error and providing sufficient power while also being computationally inexpensive. The standard F test did not control for type I error. Although it is slower (computationally) than the standard F test with ILE, the FGLS approach performed just as well as the recommended Standard F test with ILE (in terms of type I and type II error). The permutation method controlled type I error well, but did not have the same power to detect differences as the other methods.

##### PRIMARY STATISTICAL MODELS

A total of four models were run for each of the 8 intrinsic networks. All analyses used baseline imaging measures as independent variables with separate analyses being performed using baseline and 30-month assessments of DSST and eSPPB as the outcome variables. Analyses with baseline independent and dependent variables assessed simple cross-sectional associations. Analyses with baseline independent and 30-month dependent variables assessed predictive associations that imaging measures have for future cognitive and/or physical function. No covariates were included in the primary, unadjusted analyses. An adaptive false discovery rate was applied to correct for multiple comparisons across all primary analyses^72,73^ and the resultant q-values were used to for assessing statistical significance (q ⩽ 0.05).

##### SECONDARY STATISTICAL MODELS

Four sets of secondary analyses were also performed. Only brain networks that remained statistically significant after correction for multiple comparisons in the primary analyses were used in these adjusted analyses. All analyses included a measure of participant head motion in the MRI scanner as a covariate of no interest. In no analyses did this covariate meaningfully change outcomes, so it is presented in tables but not discussed in the results section. The intent of the secondary analyses was to determine if the brain network* Aβ interactions remained significant while accounting for other critical variables, such as education, age, and brain structure. One set of analyses also included the time between the completion of the main baseline study visits and the PET imaging session. Because the PET was an ancillary study, the PET scans did not begin until after the parent study was ongoing. These analyses were intended to ensure that differences in the delay to get the PET scan did not have a major effect on the outcomes. The final set of analyses was a sensitivity analysis performed to address the fact that there were different numbers of participants in the primary analyses due to missing cognitive or physical function scores at baseline or follow-up. At baseline, there were 79 participants with eSPPB scores and 81 with DSST scores. At 30-months there were 77 with eSPPB scores and 78 with DSST scores. The sensitivity analyses included 76 of the total 81 participants who had DSST and eSPPB scores at baseline and at 30 months. The analysis included education as a covariate in addition to age and head motion. All secondary analyses report nominal p-values.

##### POST-HOC ASSESSMENT OF THE DIRECTION OF SIGNIFICANT INTERACTIONS

It is, admittedly, not trivial to examine interactions with distance regression because values are unsigned pair-wise distances. Thus, to visually depict relationships of significant moderation effects, we evaluated dependent outcomes (eSPPB/DSST scores) stratified by high/low network integrity and high/low amyloid burden. The top and bottom quartiles based on network integrity were selected. To quantify community structure, the Euclidean distance between each participant’s subnetwork SI image and the *a priori* network template was computed. Individuals with higher community structure integrity would be expected to have a community that more closely matched the *a priori* template, resulting in low Euclidean distance. Within each of the top and bottom network quartile groups, the data were median split based on amyloid centiloid score. This yielded the four groupings for each network. For the CEN there were 10 participants in each of the four groups. For the SCN, the groups were not perfectly balanced with 9 high SCN/low Aβ, 10 high SCN/high Aβ, 8 low SCN/low Aβ, and 12 low SCN/low Aβ participants. Actual eSPPB and DSST scores, not distances from the regression analyses, were compared between the groupings to determine the direction of significant effects. Group average SI subnetwork maps and Aβ scores were also computed. The results depicted here were not adjusted for other covariates, comparable to results presented in Table 2. Group brain images are displayed using MRIcro software.^74^ It is important to stress that all analyses and interpretations were based on the regression analyses that utilized continuous variables for network integrity and amyloid deposition across all participants.

## RESULTS

### PARTICIPANTS

The demographic details of the study participants are presented in Table 1. The measures for the PET cohort were compared to the remainder of the study participants that were not in the PET cohort. There were no differences between groups for the variables assessed, indicating that the PET sample is representative of the groups.

### COGNITIVE AND PHYSICAL FUNCTION SCORES

Summary statistics of performance on the DSST and eSPPB are shown in Table 1. At baseline, the population had an average DSST score of 54.7 and an average eSPPB score of 2.01. The population means did not change meaningfully at 30 months, with an average DSST score of 55.4 and an average eSPPB score of 2.01. The distributions for both measures were normal at baseline (DSST: KS = 0.08, p>0.1, eSPPB: KS = 0.07, p>0.1) and at 30 months (30m DSST: KS = 0.07, p>0.1, 30m eSPPB: KS = 0.09, p>0.1).

### A*β* ASSOCIATIONS WITH COGNITIVE AND PHYSICAL FUNCTION

**Figure 2.**
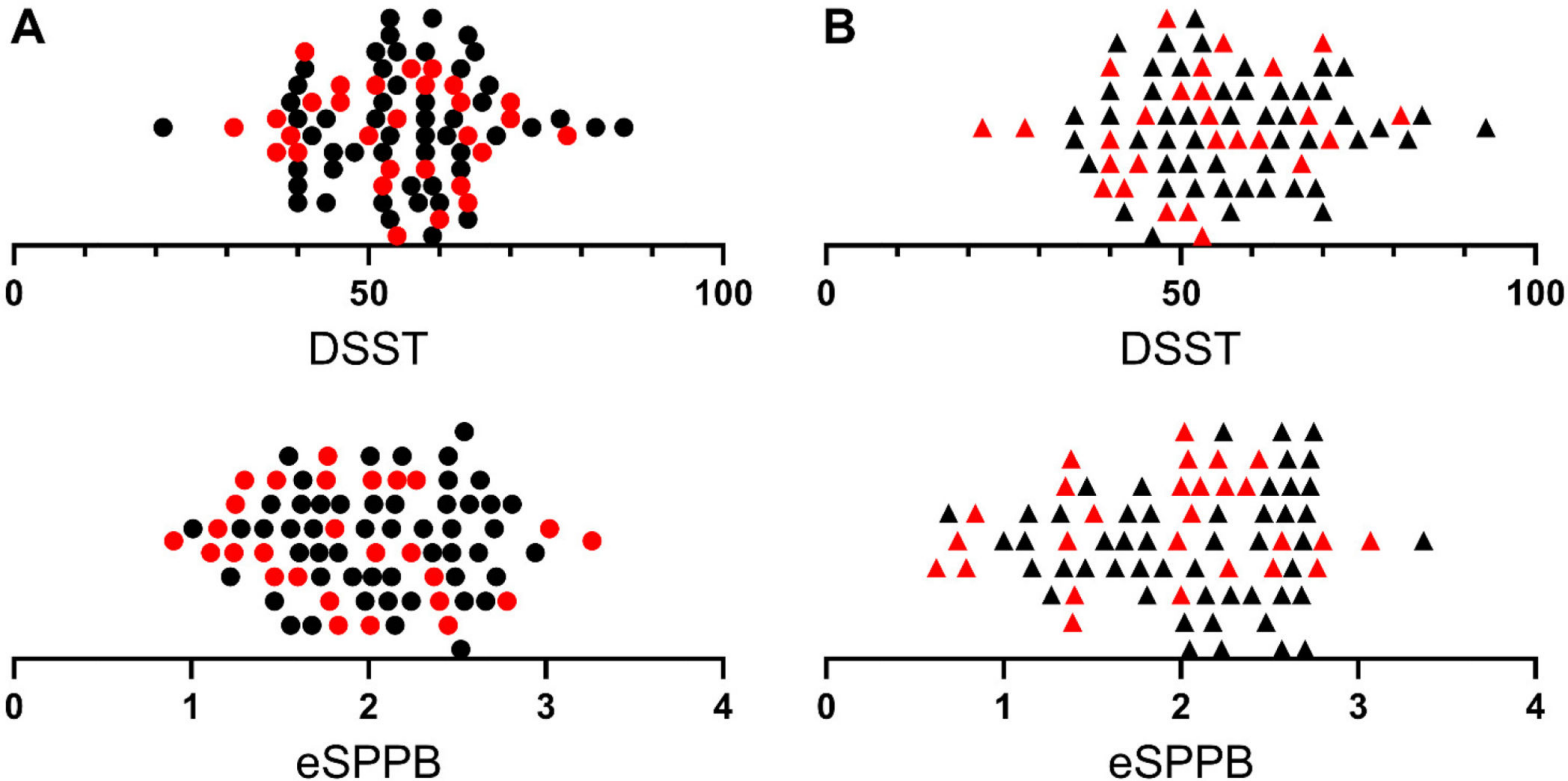
Distribution of participant DSST and eSPPB scores at baseline (A) and 30 months (B). Participants with positive Aβ scans are red. Note the Aβ positive individuals are not concentrated at the lower ends of the distribution but are represented throughout the score ranges at baseline and 30 months. Individual participant data is represented by circles at baseline and triangles at 30 months. Data points were algorithmically spread vertically for visualization purposes.

Should high brain amyloid be associated with assessments of cognitive and/or physical function, it would be expected that those individuals with higher Aβ levels would have poorer scores. In this population, such a pattern was not seen at baseline or at 30 months (Figure 2). It is clear from the figure that Aβ positive individuals are represented throughout the distribution of scores for both DSST and eSPPB at both time points. In fact, individuals that had Aβ level high enough to be labeled as clinically positive group (CL>24) had an average DSST and eSPPB within 1 standard deviation of the mean for the entire population at baseline (DSST:53.9, eSPPB: 1.88) and 30 months (DSST:51.9, eSPPB: 1.88). The Aβ positive individuals are depicted in red in Figure 2 for visualization with quantitative assessments between Aβ and function presented below.

**Figure 3.**
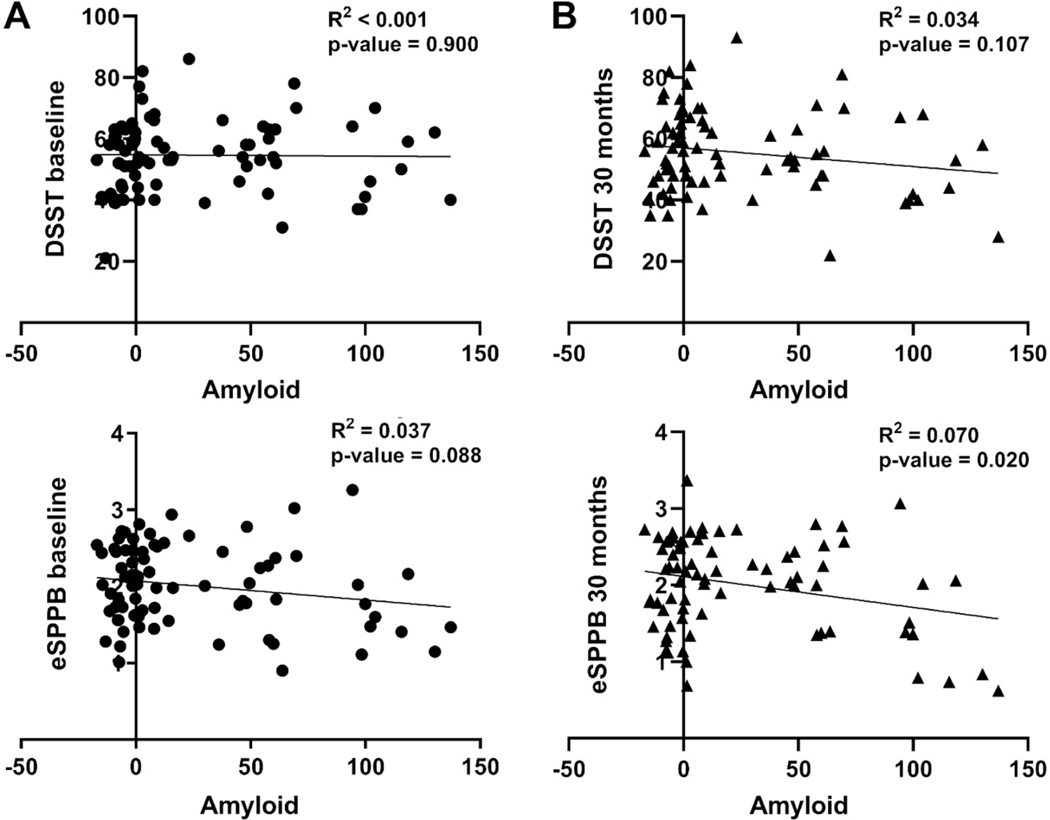
Associations of Aβ with DSST and eSPPB scores at baseline (A) and 30 months (B). Due to missing data, there are different numbers of participants in each analysis. For DSST there were 81 at baseline and 78 at 30 months. For eSPPB there were 79 at baseline and 77 at 30 months. Individual participant data is represented by circles at baseline and triangles at 30 months.

Correlations between whole-brain Aβ levels and eSPPB and DSST revealed no significant association at baseline for either measure (Figure 3A). There was a slight negative slope for eSPPB (i.e., higher Aβ may be related to lower eSPPB scores) that didn’t reach significance; we note that most of the individuals with Aβ levels near 100 CL had eSPPB scores close to the group mean. An individual with CL = 94 actually had the highest eSPPB score of the population. For DSST scores recorded at the 30-month follow-up (Figure 3B), there was a slightly negative relationship with Aβ levels, but it was not significant. The relationship between 30-month eSPPB and Aβ achieved significance but was still relatively weak, with a slope of −0.004, and this relationship only accounted for 7% of the variance in eSPPB scores.

### BRAIN NETWORK*A*β* STATISTICAL MODERATION ANALYSES

Moderation between the community structure integrity of the 8 intrinsic brain networks and Aβ levels on the outcome variables (DSST and eSPPB) were assessed using statistical interactions. Of the eight networks tested, the CEN and SCN were the two that exhibited significant interactions with Aβ for both DSST and eSPPB with correction for multiple comparisons (Table 2). All regression estimates for CEN, SCN, and amyloid that are listed in Table 2 are those observed when the interaction was present in the model. The results demonstrate that interactions consistent with cognitive reserve were present at baseline for eSPPB but were not present until the 30-month follow-up for the DSST. For the eSPPB, the CEN and SCN interactions were both significant at baseline with similar effect sizes. The CEN remained significant at 30 months, but the SCN was not. For the DSST, there were no significant interactions at baseline. However, the main effects for both the CEN and SCN were significant at baseline. At 30 months, the interactions were significant for both networks with comparable effect sizes that were ~3 times greater than observed at baseline. For completeness, results for the networks that did not exhibit significant main or interaction effects are in the supplement materials (Table S1).

### POST-HOC ASSESSMENT OF THE DIRECTION OF SIGNIFICANT INTERACTIONS

In all analyses with significant main or interaction effects, the estimates for the main or interaction effects were positive. This indicates that as the difference between subject brain network patterns increased, the difference in the behavioral outcome increased. However, the direction of the relationships for brain networks and Aβ with the behavioral outcomes cannot be determined directly from the estimates due to the use of absolute distances. To interpret the observed significant interactions, plots were developed to capture the four quadrants of the interactions (high network integrity/low Aβ, high network integrity/high Aβ, low network integrity/low Aβ, low network integrity/high Aβ). The high and low Aβ groupings were those above and below the median value, respectively. See methods section 2.6 for a description of how the individuals in each group were identified. Note that the statistical outcomes were based on a continuous distance regression including all available data for each outcome variable and these plots are for interpretational purposes.

**Figure 4.**
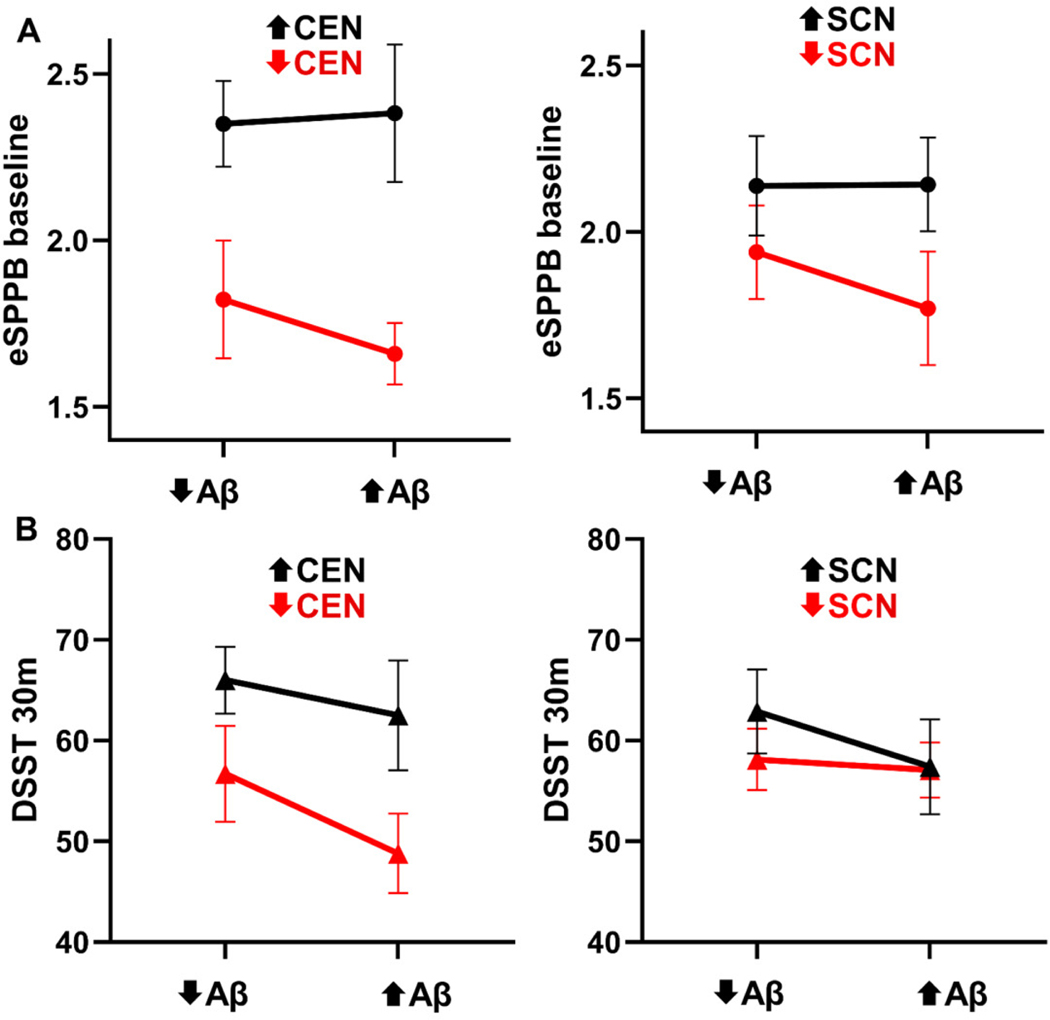
Plots depicting the direction of the significant interactions between brain network integrity and Aβ for eSPPB at baseline (A) and the DSST at 30 months (B). Plots on the left are for CEN integrity, and those on the right are for SCN integrity. The data show the mean and the standard error of the mean (SEM) for eSPPB and DSST from individuals that made up the four categorical groups and are not distances used in the regression. For network integrity, the arrows indicate the upper and lower quartiles. For amyloid levels, the arrows indicate above or below the median within the given quartile. The data in red are for individuals with low network integrity regardless Aβ level. The figure demonstrates that in all cases, low network integrity was associated with poorer performance (red line lower than black line). Differences in the slopes between the black and red lines in each plot are consistent with the significant interactions identified in the distance regressions. High Aβ had the most pronounced effect on performance in the low network integrity groups, with the SCN and DSST being the notable exceptions. All statistical analyses in the manuscript were based on the distance regression results that used continuous measures and included all study participants.

The significant interactions that the CEN and SCN had with Aβ at baseline for eSPPB and at 30 months for DSST are explored graphically in interaction plots (Figure 4). Baseline interactions for the eSPPB showed very similar relationships for the two networks. The mean eSPPB score was very similar for the low and high Aβ groups when the networks had high integrity. When networks had low integrity, the mean eSPPB score was lower overall with the high Aβ group being the lowest. Thus, high levels of Aβ were only associated with lower eSPPB scores if the networks had low integrity. The CEN* Aβ interaction for DSST at 30 months exhibited a similar pattern. Mean DSST score was highest when the networks had high integrity, with the low Aβ groups having the highest DSST. Low network integrity was associated with a lower mean DSST score in both groups with the high Aβ group having the lowest score. The SCN * Aβ interaction for DSST at 30 months was somewhat different. As observed with the CEN, the highest mean DSST score was found in participants with high network integrity and low Aβ. The mean DSST score was lower in the low network integrity/low Aβ. Those with high Aβ had a lower mean DSST score than those with low Aβ, regardless of network integrity. Figure S2 is the same data plotted with the network integrity on the x axis rather than Aβ for a different perspective of the interaction.

**Figure 5.**
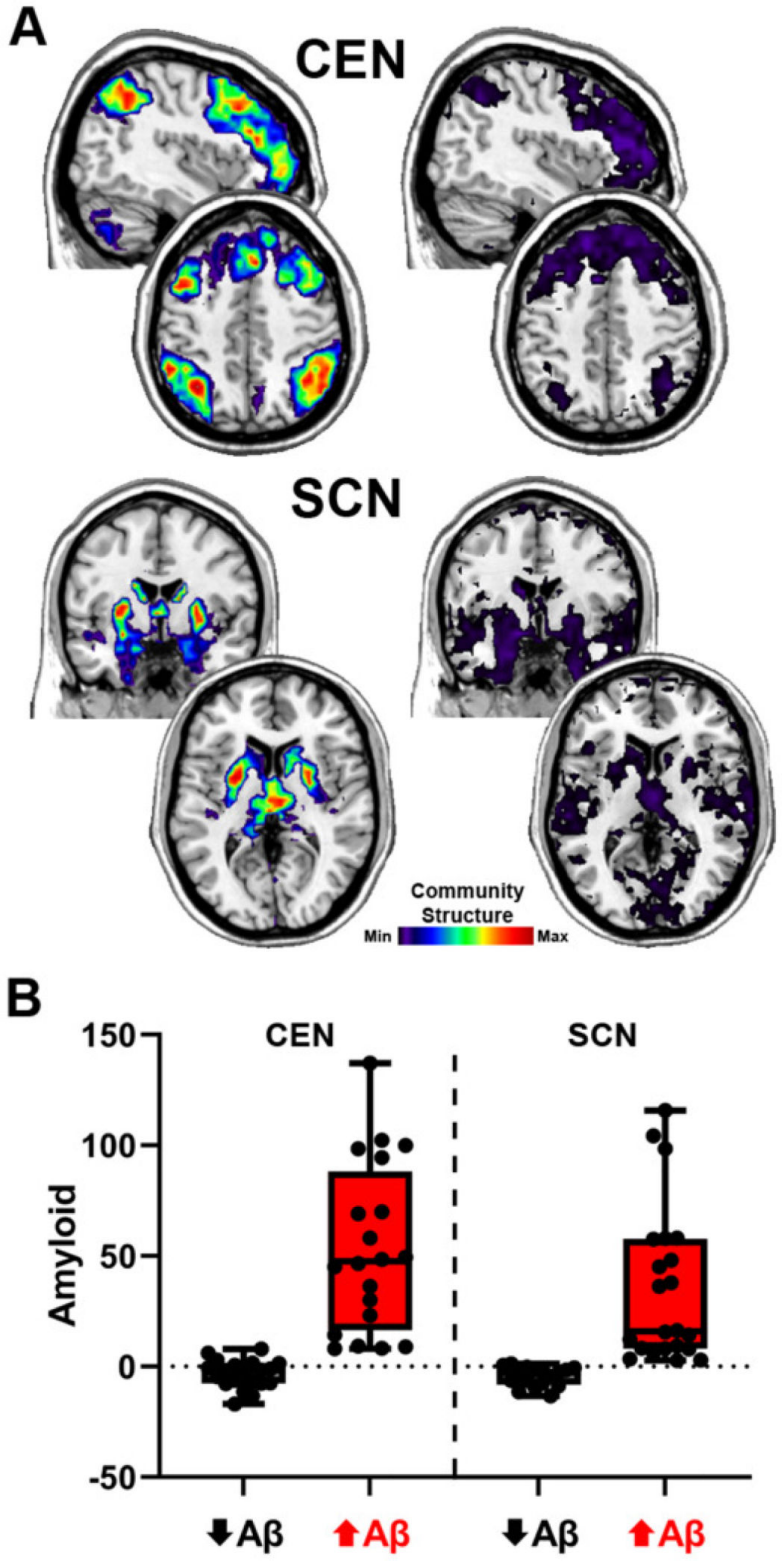
Individual data depicted for network integrity and amyloid levels to demonstrate the direction of associations identified using the distance regressions. A. Average scaled inclusivity images for the upper (left) and lower (right) quartiles for network integrity. The Montreal Neurological Institute (MNI) coordinates for the CEN images are x = 42 for the upper sagittal slice and z = 46 for the lower axial slice. Coordinates for the SCN images are y = −1 for the upper coronal slice and z = 6 for the lower axial slice. The calibration bar applies to all images, but due to the different scales, it was necessary to have a different range for each group to effectively visually depict the integrity of each network. The ranges are: CEN high (0.04–0.105) and low (0.01–0.105), and SCN high (0.02–0.05) and low (0.035–0.06), all based on scaled inclusivity as described in the methods. B. Amyloid levels based on median split for the participants included in the average network maps. Box and whisker plots for amyloid levels (centiloids) for the high and low Aβ groups (above and below the median, respectively). The participants in each grouping are different based on the quartile groupings for CEN and SCN integrity. The box depicts the boundaries of the upper and lower quartiles, the line indicates the median, and the whiskers indicate the min-max range.

The group average SI images of the CEN and SCN for the high and low network integrity groups are shown in Figure 5A. For the CEN, the spatial patterns for both groups were quite similar. The primary difference was that the magnitude of the SI was higher in the high integrity group, indicating that there was consistent spatial overlap in the CEN regions. The low SI values in the low integrity group are consistent with fragmentation of the CEN. For the SCN, not only does the low integrity group have values that are substantially lower than the high integrity group, but their map also includes regions beyond the subcortical areas scattered throughout the brain (most notably in occipital and temporal regions). This high variability in the spatial maps is consistent with SCN fragmentation as well as incorporation of other regions into communities with the SCN regions. Mean levels for the high and low Aβ groupings are shown in Figure 5B. For the CEN, the m ean values for the high and low Aβ groups were −3.4 and 52.9 centiloids, respectively. The difference between the Aβ groups was less pronounced for the SCN with means o f −4.4 and 34.8 for the low and high Aβ groups, respectively.

### SECONDARY ANALYSES

We performed secondary analyses for the CEN and SCN (Table S2) with added covariates for education and age. These additional covariates were included to account for the fact that education is often considered a proxy for CR, and the functional outcomes are known to exhibit age-related changes. Age was significant in all models, but education was not. The outcomes reported for the primary analyses all remained significant, though the estimates were lower and p-values higher.

Due to a gap between the time the MRI and PET data were collected, analyses were performed that also included the time between the completion of the baseline visits and the PET scan as well as age. Age was significant in all models. The PET scan date was significant in all models except the 30-month DSST models. Despite the significance of the covariates, there were no meaningful changes in effect sizes or significance of the primary outcomes for these analyses (Table S3).

To determine if CR associated with brain networks could be accounted for by structural brain measures, analyses were performed with additional covariates for: 1) total gray and white matter volume, 2) bilateral hippocampal volume, 3) bilateral thalamic volume, 4) bilateral prefrontal cortex volume, 5) intracranial volume, 6) white matter lesion volume, 7) as well as age and education. The effect sizes for the brain network* Aβ interactions were reduced but all remained significant except the SCN* Aβ interaction for the DSST at 30 months (Table S4). The effect size for this interaction was reduced by about ½. This was the weakest interaction in the primary analyses and given the effect size and the loss of degrees of freedom in the expanded model, it is not surprising that the interaction was no longer significant. Notably, the main effect of the SCN was significant in this adjusted analysis. There were multiple significant associations between brain anatomy variables and both DSST and eSPPB. For the DSST, hippocampal volume was found to be significant in all models. For eSPPB, white matter lesion volume was significant in all models. There were multiple other significant associations, but they were not consistent across time (baseline vs. 30-month models) or network (CEN vs. SCN). These significant findings are further detailed in Table S4.

Finally, a sensitivity analysis was performed as there were missing data for various models. This analysis used the same 76 participants that had complete data for all models. This analysis also included education and age as covariates. There were minor changes in effect sizes and no change in significance of the main study outcomes (Table S5).

## DISCUSSION

The current study utilized resting state functional brain networks to operationalize CR and determined their utility in moderating baseline and longitudinal associations of Aβ with speed of cognitive and physical function in older adults free of dementia at baseline. Consistent with the study hypotheses, we found significant interactions between network integrity (CEN and SCN) and Aβ for associations with cognitive and physical performance. These moderating effects, however, varied as a function of study outcome and time. For the physical function measure, the moderation was present in baseline cross-sectional analyses. For cognition, the moderation was present for the predictive analyses that used cognitive measures from the 30-month follow-up assessment. In all cases, the CEN exhibited stronger moderations than the SCN. These findings suggest that network integrity may be a possible neural mechanism for CR, as defined herein, and have protective effects against Aβ that extend to both cognitive and motor outcomes.

Prior work has shown that functional connectivity in fronto-striatal circuits,^75^ including the central executive^76–78^ and subcortical^79,80^ networks, is associated with cognition in aging. These prior studies used methods and measures that differed from our work. However, the findings are generally compatible with our results and appear to converge on an interpretation that less integrated connectivity^77,79^ and greater variability of activity^76^ within regions that make up the CEN and SCN are associated with poorer cognitive performance. However, higher specific fronto-striatial connectivity was shown to be associated with better cognition.^75^ Such increases in connectivity between the CEN (fronto) and SCN (striatal) would be expected to lead to declines in within-network community structure. We did not observe this in our work, but future studies could examine both measures to clarify this discrepancy.

We found that, at baseline, higher integrity of both networks was directly associated (main effect of network) with better DSST performance, a measure of speed of processing, but there were no associations between Aβ and DSST at baseline. For cognitive performance at the 30-month follow-up, there were significant interactions wherein higher integrity in both networks was protective against higher Aβ concentrations. These findings provide the first evidence that CR, operationalized via measures of functional brain network integrity, moderates associations of Aβ with future cognition in dementia-free older adults. Discrepancies between cross-sectional and longitudinal associations are common^81^; and in the current study non-significant moderation effects (i.e., interactions) between network connectivity and Aβ at baseline may be attributed to reduced variance on DSST performance given the healthy nature of this sample.

Literature concerning cognitive^8–10^ and brain^82–84^ control of physical function in aging is robust. However, knowledge concerning the role of resting state networks in physical function is relatively scarce in older adults.^37^ Notably, a recent study revealed that connectivity in the frontoparietal network is implicated in motor skill learning.^85^ Our study extends previous findings, providing first evidence that integrity in both the CEN and SCN moderated associations between Aβ and eSPPB performance at baseline. The moderation between network integrity and Aβ levels at 30-month follow-up was only significant for the CEN. This suggests that higher integrity in a network implicated in higher-order cognitive processing served as a buffer against poor current and future physical performance. Recent studies indicate that higher CR is related to more efficient brain control of walking, notably under attention-demanding conditions,^25^ and also attenuates the risk of developing incident mobility impairment.^26,27^ Hence, building on the extant limited literature, the current study introduces novel evidence for the role of CR, defined using connectivity in the CEN and SCN, as a buffer against the influence of Aβ on physical function.

It may be argued that the integrity of the CEN and SCN is a proxy for a buffer that is broader than CR. That is, a construct, previously termed individual reserve,^86^ that captures collective cognitive and physical reserve capacities within a person may better account for the ability of the brain to adapt to cognitive and physical consequences of aging. This view of reserve provides an additional lens through which cognitive-motor interactions may be understood in the context of aging and age-related neurodegeneration. However, the fact that we did not see any significant interactions between Aβ and other networks, such as the DMN, suggests that the findings are network-specific.

The cross-sectional nature of the statistical analyses used in this study are not sufficient to establish causality or unequivocally determine that brain networks moderate the effects of amyloid rather than the reverse. However, the MacArther approach to moderation analyses that is encouraged in the medical literature^67,87^ stipulates that moderators must temporally precede the exposure variable. From a mechanistic neurobiological perspective, it makes sense to ascribe the moderator role to the brain networks as a preexisting process. The amyloid deposition fits the role of the exposure variable as accumulation of amyloid is a process that occurs later in life, while brain network integrity is essential for function throughout life. Although our study was not initially designed for moderation analyses, the MacArther approach would support the “strong hypotheses”^67^ that brain networks moderate the effect of amyloid on cognitive and physical function. Future studies will need to verify the causal nature and direction of this moderating effect. If replicated, the results would indicate that brain network integrity is one of potentially many factors that provide resilience to the deposition of amyloid. This is a critical mechanistic outcome as brain network integrity could be used to assess vulnerability to amyloid and identify individuals that would most benefit from amyloid-lowering drugs.

This study is not without limitations. The study was a secondary analysis using cross-sectional data from a subset of older adult participants from the larger, parent B-NET study. The participants in the parent BNET study were specifically recruited to have normal cognition at baseline, as the primary objective was to examine brain-motor associations. It is possible that the findings observed here are specific to those individuals that exhibit normal cognition. It has been found that amyloid associations with physical function are present in individuals with, but not without, cognitive impairment.^19,20,22,88^ The field of brain network analyses is rapidly evolving and there is no standardized methodology that is universally accepted. We have chosen to use image processing and network statistical analysis methods that we have found to be robust in our prior work on older adults.^37,89–92^ From a brain mapping perspective, it is powerful to be able to demonstrate that the spatial patterns of brain network communities are related to behavioral outcomes. However, estimates from the regression technique employed can be difficult to interpret despite the fact that the method has been validated.^69^ The cognitive and physical function measures used here were selected because they are clinically relevant and related to future disability and mortality.^50–55^ However, the cognitive measure is fairly specific to processing speed and the physical function measure includes speed in 3 of the 4 assessed domains. Thus, generalizability of the findings to other cognitive and physical function domains will need to be assessed in future research performing targeted hypothesis testing based on the findings presented here. Finally, although to our knowledge this is the first study to combine brain networks and amyloid in examining associations with cognitive and physical function, the study sample is relatively small and homogeneous. It will be important to replicate our results in future studies using larger sample sizes and more diverse populations.

### CLINICAL IMPLICATIONS AND FUTURE DIRECTIONS

These findings are notable given that Aβ-targeting pharmacological agents are quite effective at lowering brain amyloid levels but have small^93–96^ or no^97^ effect on cognitive function and have significant, potentially life-threatening side effects.^98^ It has also been shown that older adults with elevated brain Aβ deposition can have normal cognition,^99,100^ as was found in more than 35% of the participants in this study. There appear to be heterogenous responses to Aβ deposition and to the Aβ reducing drugs, and it may be that brain network integrity is one of the underlying factors. Given that the current study was limited to persons with normal cognition, it is not known how Aβ and brain networks may interact in those individuals with cognitive impairment. Future studies should be specifically designed to test the moderation between brain networks and amyloid and should include individuals with cognitive impairment. Studies could also consider examining apolipoprotein E (APOE) 4 carriers and noncarriers separately or include the genotype as an additional factor. If future research confirms the moderation between brain network integrity and Aβ levels not only in normal cognition but also in cognitive decline, then it may be pertinent to assess network integrity in individuals prior to the initiation of amyloid lowering treatments.

## Supplementary Material

Laurienti_25_Supp

Download: https://apertureneuro.org/article/141046-moderation-between-resting-state-connectivity-and-brain-amyloid-levels-on-speed-of-cognitive-and-physical-function-in-older-adults-evidence-for-netwo/attachment/289735.docx

## Figures and Tables

**Table 1.** Baseline Characteristics of Participants By PET Cohort

	Not in PET Cohort (n=111)	In PET Cohort (n=81)	Overall (n=192)	P-value

Age	76.43 (4.72)	76.43 (4.75)	76.43 (4.72)	0.9970
Race/Ethnicity				0.5689
White	98 (88.3)	73 (90.1)	171 (89.1)	
African American/Black	12 (10.8)	6 (7.4)	18 (9.4)	
American Indian or Alaskan Native	1 (0.9)	1 (1.2)	2 (1.0)	
Asian	0 (0.0)	1 (1.2)	1 (0.5)	
Sex				0.1789
Men	44 (39.6)	40 (49.4)	84 (43.8)	
Women	67 (60.4)	41 (50.6)	108 (56.3)	
Years of Education	15.67 (2.20)	15.70 (2.77)	15.68 (2.45)	0.9178
PET measures				
Days from Baseline to PET Visit		260.64 (112.42)		
Centiloid Score		25.39 (40.29)		
# positive scans (CL > 24)		29		
MRI measures				
Total gray matter volume in CC	596.12 (87.05)	606.16 (70.97)	600.36 (80.61)	0.3958
Bilat. hippocampus volume in CC	7.15 (0.91)	7.31 (0.99)	7.22 (0.95)	0.2658
Bilat. thalamus volume in CC	4.60 (0.81)	4.70 (0.92)	4.64 (0.85)	0.4442
Bilat. PFC volume in CC	31.51 (4.72)	31.88 (5.20)	31.67 (4.92)	0.6054
MoCA Adjusted Score	25.68 (2.19)	25.58 (2.22)	25.64 (2.20)	0.7672
DSST	55.57 (12.62)	54.65 (11.65)	55.18 (12.20)	0.6097
eSPPB	2.00 (0.53)	2.01 (0.52) (n=79)	2.00 (0.52) (n=190)	0.8712

PET – positron emission tomography, CL – centiloid, CC – cubic centimeters, Bilat – bilateral, MoCA - Montreal Cognitive Assessment, DSST – digit symbol substitution test, eSPPB – expanded short physical performance battery

**Table 2.** Regression results for the CEN and SCN

Outcome Variable	Independent Variables	Estimate	SE	T score	p-Value	FDR q-Value

Baseline eSPPB	amyloid	−0.0066	0.0020	−3.2953	0.0010	0.0127
	CEN	0.8278	0.2552	3.2442	0.0012	0.0127
	amyloid*CEN	**0.0099**	**0.0028**	**3.4911**	**0.0005**	**0.0127**

	amyloid	−0.0072	0.0023	−3.0875	0.0020	0.0187
	SCN	0.1286	0.2650	0.4851	0.6276	0.8248
	amyloid*SCN	**0.0098**	**0.0030**	**3.2489**	**0.0012**	**0.0127**

Baseline DSST	amyloid	−0.0516	0.0403	−1.2806	0.2004	0.4487
	**CEN**	**24.7596**	**5.1641**	**4.7946**	**0.0000**	**0.0002**
	amyloid*CEN	0.0697	0.0571	1.2204	0.2224	0.4604

	amyloid	−0.0559	0.0465	−1.2026	0.2292	0.4604
	**SCN**	**16.0647**	**5.2288**	**3.0724**	**0.0021**	**0.0187**
	amyloid*SCN	0.0695	0.0606	1.1475	0.2513	0.4762

30-month eSPPB	amyloid	−0.0082	0.0024	−3.4125	0.0007	0.0127
	CEN	0.4014	0.3141	1.2777	0.2015	0.4487
	amyloid*CEN	**0.0127**	**0.0034**	**3.7287**	**0.0002**	**0.0094**

	amyloid	−0.0057	0.0028	−2.0233	0.0431	0.1532
	SCN	0.0555	0.3255	0.1704	0.8647	0.9697
	amyloid*SCN	0.0084	0.0037	2.2801	0.0227	0.1090

30-month DSST	amyloid	−0.1596	0.0486	−3.2854	0.0010	0.0127
	CEN	18.9454	6.3188	2.9983	0.0027	0.0219
	amyloid*CEN	0.2311	0.0687	**3.3634**	**0.0008**	**0.0127**

	amyloid	−0.1561	0.0567	−2.7510	0.0060	0.0410
	SCN	16.2112	6.5487	2.4755	0.0134	0.0715
	amyloid*SCN	**0.2072**	**0.0737**	**2.8119**	**0.0050**	**0.0366**

Significant interactions or main effects in the absence of an interaction are bolded. SE- standard error

CEN - Central Executive Network, SCN – Subcortical Network, FDR - false discovery rate

DSST - digit symbol substitution test, eSPPB - expanded short physical performance battery

## Data Availability

Data can be made available upon request to the authors with appropriate Institutional Review Board approval and data use agreements.
