# Supplementary material for: Moderation between resting-state connectivity and brain amyloid levels on speed of cognitive and physical function in older adults: Evidence for network-based cognitive reserve
Source: Apert Neuro. Author manuscript; Available in PMC 2026 Jan 16. (PMC12807530; doi:10.52294/001c.141046)
Supplement: Laurienti_25_Supp [file NIHMS2116856-supplement-Laurienti_25_Supp.pdf]

Resting-state connectivity modifies the effects of amyloid on speed of cognitive and physical function: Evidence for network-based cognitive reserve.

Supplemental Results

Supplemental Figure 1

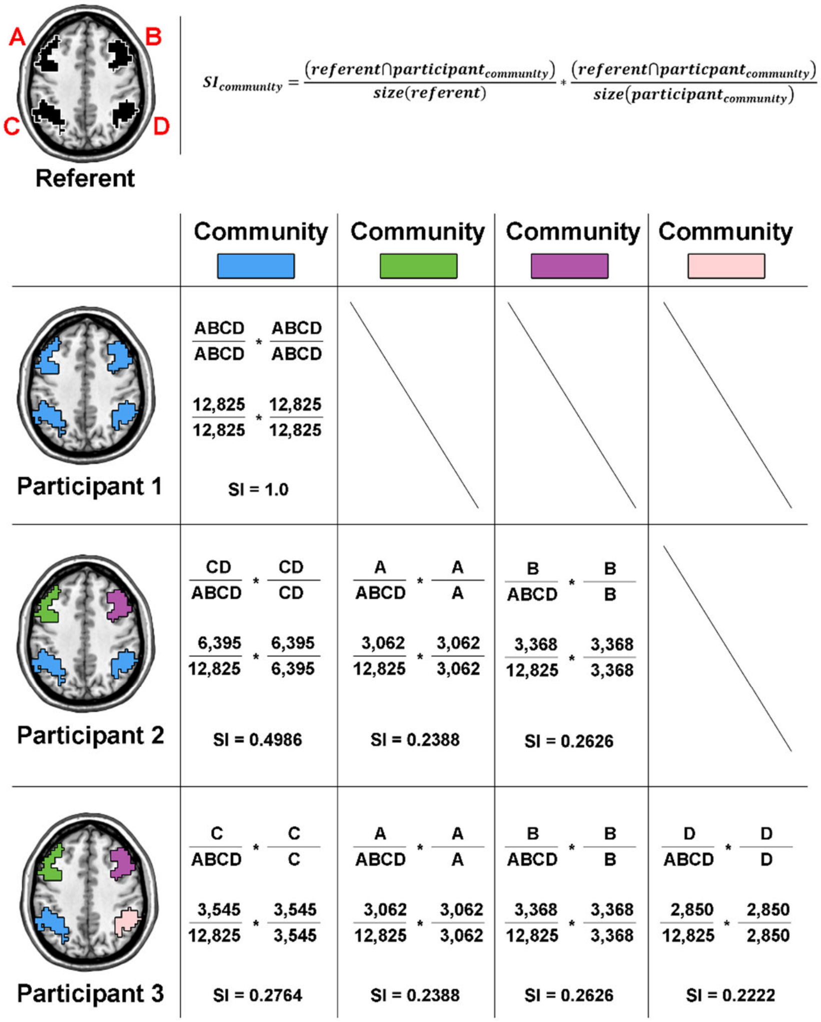

Figure S1. Scaled Inclusivity (SI) cartoon depiction. The top figure shows the referent community (regions that belong to the CEN in this example) and the equation of SI. The community structure from three hypothetical participants is shown below. Every voxel in the brain network will belong to a community, but we are only displaying hypothetical communities that overlap with referent subnetwork. All numbers in the participant-specific SI calculations represent the number of voxels in our simulated data. In Participant 1, there is a community that has perfect overlap with the referent. The voxels in this community would all be assigned an SI of 1. The SI value for voxels that belong to a community with no overlap with the referent will be assigned an SI of 0 (all other voxels in the brain in this cartoon). In Participant #2 the parietal regions of the referent (areas C and D) belong to one community while the frontal regions (A and B) each occupy individual communities. Note that the SI values for the two parietal regions are identical and are the largest because that community occupies nearly half of the referent subnetwork. In Participant #3, all four regions belong to separate communities. The voxels within each region have the same SI (ex. region C overlaps with the teal community and all voxels in that region get an SI value of 0.2764) but the four regions have distinct SI values. Due to the regions having similar sizes, the four regions have similar SI values. In this simplified cartoon we have shown examples where the network communities directly overlap with regions in the referent subnetwork. In reality, it is likely that communities will partially overlap with regions and contain nodes that are totally nonoverlapping with the subnetwork. Thus, regions outside of the referent network will also have non-zero SI values.

Supplemental Figure 2

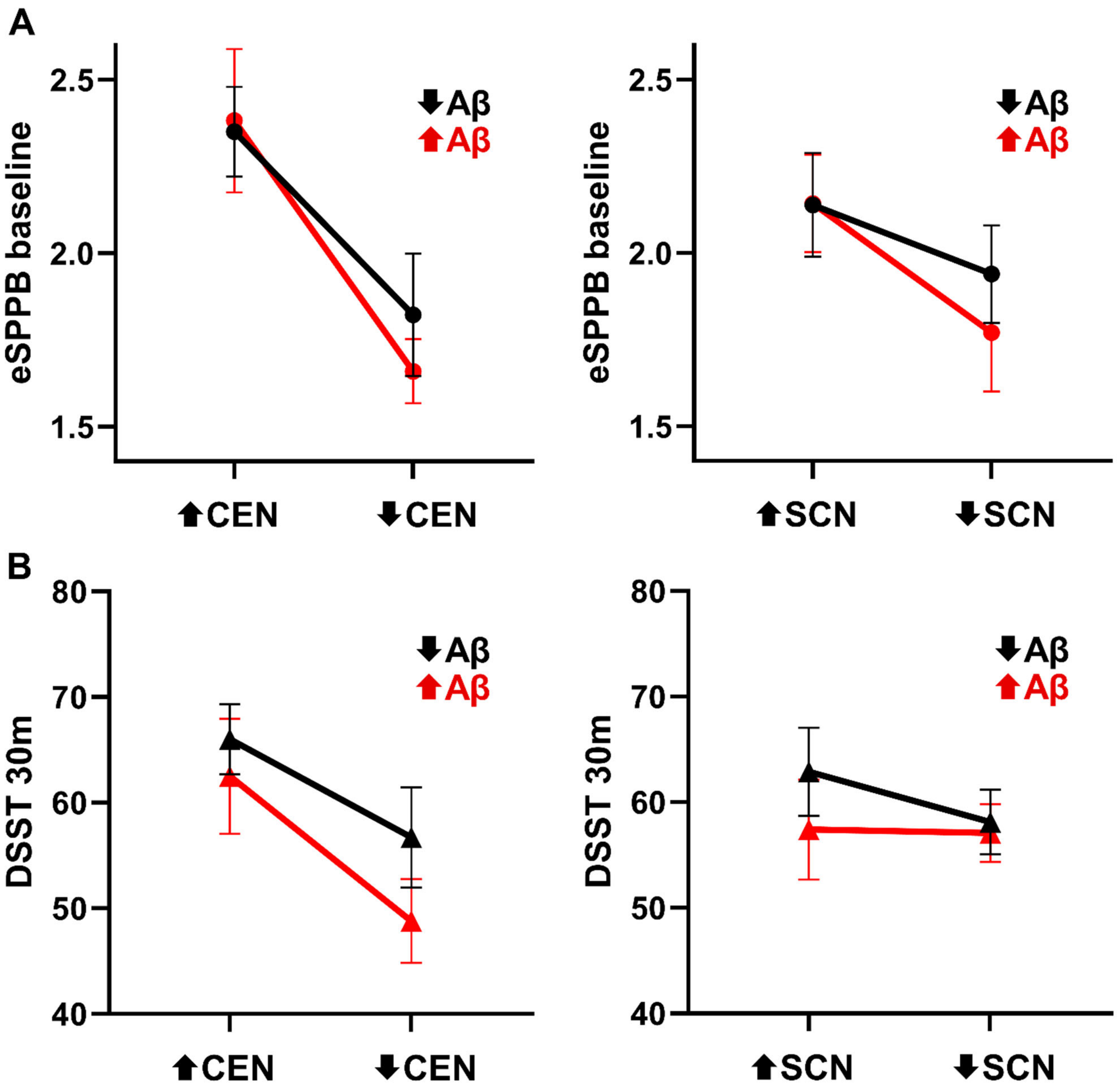

Figure S2. Plots depicting the direction of the significant interactions between brain networks and Aβ for eSPPB at baseline (A) and the DSST at 30 months (B). These plots have networks on the x axis and Aβ in red and black as opposed to Figure 3 in the manuscript. The figure is shown for a different perspective of the interaction. The data show the mean and the standard error of the mean (SEM) for the four categorical groups. For network integrity, the ⬆/⬇ indicate the upper and lower quartiles. For amyloid levels, the ⬆/⬇ indicate above or below the median within the given quartile. The data in red are for individuals with high amyloid regardless of network integrity. The figure demonstrates the direction of the relationships and interaction between network integrity/Aβ on behavioral test scores. All quantitative assessments in the manuscript were based on the regression results that used continuous measures and included all study participants.

Table S1. Model results for networks without significant findings

Outcome Variable	Independent Variables	Estimate	SE	T score	FDR p-Value	Outcome Variable	Independent Variables	Estimate	SE	T score	FDR p-Value
DAN											
Baseline DSST	amyloid	-0.0858	0.0597	-1.4375	0.3918	30-month DSST	amyloid	-0.0850	0.0711	-1.1941	0.4604
	DAN	-2.3657	5.0782	-0.4659	0.8248		DAN	0.0238	6.1144	0.0039	0.9980
	amyloid*DAN	0.1062	0.0763	1.3912	0.4143		amyloid*DAN	0.1120	0.0909	1.2316	0.4604
Baseline eSPPB	amyloid	0.0005	0.0030	0.1575	0.9697	30-month eSPPB	amyloid	-0.0007	0.0035	-0.2068	0.9697
	DAN	-0.1628	0.2520	-0.6461	0.7290		DAN	-0.3087	0.3084	-1.0009	0.5339
	amyloid*DAN	-0.0002	0.0038	-0.0457	0.9980		amyloid*DAN	0.0018	0.0045	0.4025	0.8455
DMN											
Baseline DSST	amyloid	-0.0864	0.0540	-1.6003	0.3300	30-month DSST	amyloid	-0.1357	0.0654	-2.0751	0.1407
	DMN	6.8118	5.2159	1.3060	0.4487		DMN	3.5836	6.3282	0.5663	0.7721
	amyloid*DMN	0.1121	0.0722	1.5524	0.3417		amyloid*DMN	0.1852	0.0874	2.1196	0.1363
Baseline eSPPB	amyloid	-0.0042	0.0027	-1.5628	0.3417	30-month eSPPB	amyloid	-0.0035	0.0032	-1.0859	0.5132
	DMN	-0.1649	0.2587	-0.6376	0.7290		DMN	-0.1911	0.3135	-0.6096	0.7433
	amyloid*DMN	0.0061	0.0036	1.6935	0.2896		amyloid*DMN	0.0056	0.0043	1.3039	0.4487
FTN											
Baseline DSST	amyloid	-0.1243	0.0578	-2.1512	0.1363	30-month DSST	amyloid	-0.1778	0.0687	-2.5898	0.0545
	FTN	10.8537	6.5752	1.6507	0.3063		FTN	-7.1411	7.8908	-0.9050	0.5608
	amyloid*FTN	0.1561	0.0742	2.1031	0.1363		amyloid*FTN	0.2320	0.0881	2.6330	0.0529
Baseline eSPPB	amyloid	0.0004	0.0029	0.1315	0.9697	30-month eSPPB	amyloid	-0.0006	0.0034	-0.1632	0.9697
	FTN	-0.0048	0.3269	-0.0147	0.9980		FTN	-0.1778	0.3948	-0.4503	0.8248
	amyloid*FTN	-0.0001	0.0037	-0.0161	0.9980		amyloid*FTN	0.0016	0.0044	0.3672	0.8677

DAN - dorsal attention network, DMN - default mode network, FTN - frontotemporal network, SMN - sensorimotor network, SN - salience network, VN - visual network

DSST - digit symbol substitution test, eSPPB - extended short physical performance battery, SE - standard error, FDR - false discovery rate

Table S1 cont. Model results for networks without significant findings

Outcome Variable	Independent Variables	Estimate	SE	T score	FDR p-Value	Outcome Variable	Independent Variables	Estimate	SE	T score	FDR p-Value
SMN											
Baseline DSST	amyloid	-0.0132	0.0540	-0.2440	0.9684	30-month DSST	amyloid	-0.0600	0.0652	-0.9204	0.5608
	SMN	1.0788	5.5019	0.1961	0.9697		SMN	-6.8361	6.6784	-1.0236	0.5339
	amyloid*SMN	0.0133	0.0704	0.1886	0.9697		amyloid*SMN	0.0816	0.0849	0.9616	0.5472
Baseline eSPPB	amyloid	0.0000	0.0027	-0.0026	0.9980	30-month eSPPB	amyloid	0.0028	0.0033	0.8717	0.5745
	SMN	-0.1219	0.2713	-0.4492	0.8248		SMN	-0.1419	0.3339	-0.4248	0.8366
	amyloid*SMN	0.0004	0.0035	0.1275	0.9697		amyloid*SMN	-0.0028	0.0042	-0.6618	0.7279
SN											
Baseline DSST	amyloid	-0.0418	0.0435	-0.9594	0.5472	30-month DSST	amyloid	-0.1111	0.0524	-2.1210	0.1363
	SN	8.3739	5.4680	1.5314	0.3456		SN	-0.0560	6.5687	-0.0085	0.9980
	amyloid*SN	0.0538	0.0598	0.9002	0.5608		amyloid*SN	0.1566	0.0718	2.1820	0.1335
Baseline eSPPB	amyloid	-0.0040	0.0022	-1.8351	0.2205	30-month eSPPB	amyloid	-0.0062	0.0026	-2.3429	0.0970
	SN	-0.3634	0.2703	-1.3446	0.4406		SN	-0.2806	0.3277	-0.8563	0.5790
	amyloid*SN	0.0059	0.0030	1.9987	0.1567		amyloid*SN	0.0094	0.0036	2.6213	0.0529
VN											
Baseline DSST	amyloid	-0.0555	0.0526	-1.0540	0.5289	30-month DSST	amyloid	-0.0718	0.0627	-1.1440	0.4762
	VN	-4.0989	4.3095	-0.9511	0.5472		VN	-2.4705	5.1611	-0.4787	0.8248
	amyloid*VN	0.0641	0.0641	1.0000	0.5339		amyloid*VN	0.0907	0.0763	1.1883	0.4604
Baseline eSPPB	amyloid	0.0000	0.0026	-0.0101	0.9980	30-month eSPPB	amyloid	-0.0033	0.0031	-1.0365	0.5333
	VN	-0.1526	0.2148	-0.7106	0.6938		VN	-0.3848	0.2592	-1.4846	0.3680
	amyloid*VN	0.0004	0.0032	0.1363	0.9697		amyloid*VN	0.0048	0.0038	1.2599	0.4538

DAN - dorsal attention network, DMN - default mode network, FTN - frontotemporal network, SMN - sensorimotor network, SN - salience network, VN - visual network

DSST - digit symbol substitution test, eSPPB - extended short physical performance battery, SE - standard error, FDR - false discovery rate

Table S2. Adjusted models including education and head motion

Outcome Variable	Independent Variables	Estimate	SE	T score	p-Value	Outcome Variable	Independent Variables	Estimate	SE	T score	p-Value
SCN											
Baseline DSST	amyloid	-0.0424	0.0465	-0.9107	0.3625	30-month DSST	amyloid	-0.1300	0.0565	-2.3005	0.0215
	education	0.0191	0.0673	0.2835	0.7768		education	0.0237	0.0811	0.2920	0.7703
	**age**	**0.1550**	**0.0393**	**3.9438**	**0.0001**		**age**	**0.3027**	**0.0462**	**6.5525**	**<0.0001**
	head motion	0.0098	0.0223	0.4392	0.6605		head motion	-0.0099	0.0263	-0.3766	0.7065
	**SCN**	**15.4126**	**5.2287**	**2.9477**	**0.0032**		SCN	14.9455	6.5211	2.2919	0.0220
	amyloid*SCN	0.0507	0.0606	0.8355	0.4035		**amyloid*SCN**	**0.1708**	**0.0734**	**2.3255**	**0.0201**
Baseline eSPPB	amyloid	-0.0052	0.0023	-2.2749	0.0230	30-month eSPPB	amyloid	-0.0039	0.0028	-1.3885	0.1651
	education	0.0023	0.0033	0.6936	0.4880		education	-0.0018	0.0040	-0.4444	0.6568
	**age**	**0.0207**	**0.0019**	**10.8418**	**<0.0001**		**age**	**0.0196**	**0.0023**	**8.5233**	**<0.0001**
	**head motion**	0.0027	0.0011	2.4916	0.0128		head motion	0.0016	0.0013	1.2059	0.2279
	SCN	-0.0014	0.2606	-0.0052	0.9958		SCN	-0.0741	0.3224	-0.2299	0.8182
	**amyloid*SCN**	**0.0071**	**0.0030**	**2.3775**	**0.0175**		**amyloid*SCN**	0.0058	0.0036	1.6032	0.1090
CEN											
Baseline DSST	amyloid	-0.0427	0.0403	-1.0593	0.2895	30-month DSST	amyloid	-0.1416	0.0483	-2.9289	0.0034
	education	0.0107	0.0671	0.1602	0.8727		education	0.0191	0.0808	0.2369	0.8128
	**age**	**0.1533**	**0.0391**	**3.9213**	**0.0001**		**age**	**0.3016**	**0.0460**	**6.5582**	**<0.0001**
	head motion	-0.0032	0.0224	-0.1425	0.8867		head motion	-0.0243	0.0266	-0.9137	0.3609
	**CEN**	**24.5945**	**5.1880**	**4.7407**	**<0.0001**		CEN	18.9360	6.3346	2.9893	0.0028
	amyloid*CEN	0.0558	0.0572	0.9759	0.3292		**amyloid*CEN**	**0.2029**	**0.0684**	**2.9650**	**0.0031**
Baseline eSPPB	amyloid	-0.0053	0.0020	-2.6961	0.0071	30-month eSPPB	amyloid	-0.0070	0.0024	-2.9211	0.0035
	education	0.0023	0.0033	0.7070	0.4796		education	-0.0016	0.0040	-0.4016	0.6880
	**age**	**0.0204**	**0.0019**	**10.7515**	**<0.0001**		**age**	**0.0191**	**0.0023**	**8.3407**	**<0.0001**
	head motion	0.0020	0.0011	1.8538	0.0639		head motion	0.0009	0.0013	0.7256	0.4681
	CEN	0.7402	0.2521	2.9360	0.0033		CEN	0.3177	0.3136	1.0132	0.3111
	**amyloid*CEN**	**0.0079**	**0.0028**	**2.8253**	**0.0048**		**amyloid*CEN**	**0.0108**	**0.0034**	**3.1886**	**0.0014**

Significant interactions or main effects in the absence of an interaction are bolded.

CEN - Central Executive Network, SCN - Subcortical Network, DSST - digit symbol substitution test, eSPPB - expanded short physical performance battery

SE- standard error

Table S3. Adjusted models including age, PET date, and head motion

Outcome Variable	Independent Variables	Estimate	SE	T score	p-Value	Outcome Variable	Independent Variables	Estimate	SE	T score	p-Value
SCN											
Baseline DSST	amyloid	-0.0420	0.0465	-0.9030	0.3666	30-month DSST	amyloid	-0.1298	0.0565	-2.2973	0.0217
	**age**	**0.1550**	**0.0392**	**3.9491**	**0.0001**		**age**	**0.3023**	**0.0462**	**6.5471**	**<0.0001**
	**PET date**	**0.0070**	**0.0022**	**3.1470**	**0.0017**		PET date	0.0050	0.0029	1.7064	0.0880
	head motion	0.0105	0.0223	0.4709	0.6377		head motion	-0.0096	0.0263	-0.3644	0.7156
	**SCN**	**15.5554**	**5.2200**	**2.9800**	**0.0029**		SCN	15.0364	6.5166	2.3074	0.0211
	amyloid*SCN	0.0504	0.0605	0.8323	0.4053		**amyloid*SCN**	**0.1706**	**0.0734**	**2.3245**	**0.0202**
Baseline eSPPB	amyloid	-0.0052	0.0023	-2.2641	0.0236	30-month eSPPB	amyloid	-0.0039	0.0028	-1.3877	0.1653
	**age**	**0.0207**	**0.0019**	**10.8725**	**<0.0001**		**age**	**0.0196**	**0.0023**	**8.5293**	**<0.0001**
	**PET date**	**0.0005**	**0.0001**	**4.3942**	**<0.0001**		**PET date**	**0.0003**	**0.0001**	**2.0323**	**0.0422**
	**head motion**	**0.0027**	**0.0011**	**2.5502**	**0.0108**		head motion	0.0016	0.0013	1.2219	0.2218
	SCN	0.0031	0.2597	0.0120	0.9905		SCN	-0.0632	0.3221	-0.1962	0.8445
	**amyloid*SCN**	**0.0070**	**0.0030**	**2.3731**	**0.0177**		amyloid*SCN	0.0058	0.0036	1.6054	0.1085
CEN											
Baseline DSST	amyloid	-0.0478	0.0403	-1.1870	0.2353	30-month DSST	amyloid	-0.1444	0.0484	-2.9862	0.0028
	**age**	**0.1533**	**0.0390**	**3.9267**	**0.0001**		**age**	**0.3013**	**0.0460**	**6.5555**	**<0.0001**
	**PET date**	**0.0064**	**0.0022**	**2.8885**	**0.0039**		PET date	0.0044	0.0029	1.5147	0.1300
	head motion	-0.0022	0.0224	-0.0965	0.9232		head motion	-0.0236	0.0265	-0.8896	0.3737
	**CEN**	**23.6575**	**5.1904**	**4.5579**	**<0.0001**		CEN	18.2942	6.3466	2.8825	0.0040
	amyloid*CEN	0.0634	0.0572	1.1091	0.2675		**amyloid*CEN**	**0.2070**	**0.0684**	**3.0246**	**0.0025**
Baseline eSPPB	amyloid	-0.0056	0.0020	-2.8728	0.0041	30-month eSPPB	amyloid	-0.0072	0.0024	-3.0195	0.0026
	**age**	**0.0204**	**0.0019**	**10.7800**	**<0.0001**		**age**	**0.0191**	**0.0023**	**8.3501**	**<0.0001**
	**PET date**	**0.0005**	**0.0001**	**4.2927**	**<0.0001**		**PET date**	**0.0003**	**0.0001**	**2.0139**	**0.0441**
	head motion	0.0021	0.0011	1.9366	0.0529		head motion	0.0010	0.0013	0.7617	0.4463
	CEN	0.6711	0.2519	2.6644	0.0078		CEN	0.2703	0.3141	0.8603	0.3897
	**amyloid*CEN**	**0.0084**	**0.0028**	**3.0099**	**0.0026**		**amyloid*CEN**	**0.0111**	**0.0034**	**3.2906**	**0.0010**

Significant interactions or main effects in the absence of an interaction are bolded.

CEN - Central Executive Network, SCN - Subcortical Network, DSST - digit symbol substitution test, eSPPB - expanded short physical performance battery

SE- standard error

Table S4. Adjusted models including age, brain volume measures, education, and head motion

Outcome Variable	Independent Variables	Estimate	SE	T score	p-Value	Outcome Variable	Independent Variables	Estimate	SE	T score	p-Value
SCN											
Baseline DSST	amyloid	-0.0155	0.0460	-0.3364	0.7366	30-month DSST	amyloid	-0.0925	0.0559	-1.6554	0.0979
	**age**	**0.1245**	**0.0389**	**3.1999**	**0.0014**		**age**	**0.2660**	**0.0458**	**5.8125**	**<0.0001**
	gm_vol	0.0017	0.0043	0.4101	0.6818		**gm_vol**	**-0.0111**	**0.0051**	**-2.1814**	**0.0292**
	**wm_vol**	**0.0145**	**0.0050**	**2.9203**	**0.0035**		wm_vol	0.0032	0.0061	0.5189	0.6039
	**bilat_hippo**	**0.9144**	**0.2517**	**3.6324**	**0.0003**		**bilat_hippo**	**2.2556**	**0.3033**	**7.4360**	**<0.0001**
	**bilat_thal**	**0.9547**	**0.2111**	**4.5230**	**<0.0001**		bilat_thal	0.3136	0.2546	1.2319	0.2181
	bilat_pfc	0.0226	0.0519	0.4358	0.6630		bilat_pfc	0.0649	0.0617	1.0521	0.2928
	**ICV**	**-0.0043**	**0.0014**	**-3.0475**	**0.0023**		ICV	-0.0016	0.0017	-0.9692	0.3325
	**WMLV**	**0.0961**	**0.0316**	**3.0384**	**0.0024**		WMLV	0.0601	0.0377	1.5950	0.1108
	education	0.0393	0.0666	0.5900	0.5552		education	0.0397	0.0802	0.4955	0.6203
	head motion	0.0034	0.0221	0.1531	0.8784		head motion	-0.0119	0.0261	-0.4553	0.6489
	**SCN**	**15.8854**	**5.1561**	**3.0809**	**0.0021**		**SCN**	**16.2188**	**6.4317**	**2.5217**	**0.0117**
	amyloid*SCN	0.0134	0.0600	0.2241	0.8227		amyloid*SCN	0.1172	0.0726	1.6136	0.1067
Baseline eSPPB	amyloid	-0.0049	0.0023	-2.1498	0.0317	30-month eSPPB	amyloid	-0.0036	0.0028	-1.2876	0.1980
	**age**	**0.0203**	**0.0019**	**10.6001**	**<0.0001**		**age**	**0.0192**	**0.0023**	**8.3278**	**<0.0001**
	gm_vol	0.0002	0.0002	0.7498	0.4534		**gm_vol**	**0.0009**	**0.0003**	**3.3269**	**0.0009**
	wm_vol	0.0000	0.0003	-0.0042	0.9967		wm_vol	0.0001	0.0003	0.2513	0.8016
	bilat_hippo	0.0077	0.0126	0.6080	0.5432		bilat_hippo	-0.0186	0.0154	-1.2081	0.2271
	bilat_thal	0.0024	0.0106	0.2262	0.8210		bilat_thal	-0.0136	0.0128	-1.0615	0.2885
	bilat_pfc	-0.0045	0.0026	-1.7268	0.0843		**bilat_pfc**	**-0.0076**	**0.0031**	**-2.4295**	**0.0152**
	ICV	0.0000	0.0001	0.3880	0.6981		ICV	-0.0001	0.0001	-0.9972	0.3187
	**WMLV**	**0.0040**	**0.0016**	**2.5391**	**0.0112**		**WMLV**	**0.0077**	**0.0021**	**3.5662**	**0.0004**
	education	0.0024	0.0033	0.7157	0.4743		education	-0.0016	0.0040	-0.3910	0.6958
	**head motion**	**0.0025**	**0.0011**	**2.3397**	**0.0194**		head motion	0.0011	0.0013	0.8487	0.3961
	**SCN**	-0.0001	0.2606	-0.0003	0.9997		SCN	-0.0720	0.3217	-0.2238	0.8229
	**amyloid*SCN**	**0.0067**	**0.0030**	**2.2464**	**0.0247**		amyloid*SCN	0.0055	0.0036	1.4998	0.1338

Significant interactions or main effects in the absence of an interaction are bolded.

CEN - Central Executive Network, SCN - Subcortical Network, DSST - digit symbol substitution test, eSPPB - expanded short physical performance battery

gm_vol - gray matter volume, wm_vol - white matter volume, bilat_hippo - bilateral hippocampal volume, bilat_thal - bilateral thalamus volume

bilat_pfc - bilateral prefrontal cortex volume, ICV - intracranial volume, WMLV - what matter lesion volume, SE- standard error

Table S4 cont. Adjusted models including age, brain volume measures, education, and head motion

Outcome Variable	Independent Variables	Estimate	SE	T score	p-Value	Outcome Variable	Independent Variables	Estimate	SE	T score	p-Value
CEN											
Baseline DSST	amyloid	-0.0248	0.0399	-0.6206	0.5349	30-month DSST	amyloid	-0.1173	0.0478	-2.4534	0.0142
	**age**	**0.1241**	**0.0387**	**3.2051**	**0.0014**		**age**	**0.2658**	**0.0456**	**5.8323**	**<0.0001**
	gm_vol	0.0002	0.0043	0.0477	0.9619		**gm_vol**	**-0.0126**	**0.0051**	**-2.4715**	**0.0135**
	**wm_vol**	**0.0135**	**0.0049**	**2.7276**	**0.0064**		wm_vol	0.0019	0.0061	0.3107	0.7560
	**bilat_hippo**	**0.9453**	**0.2508**	**3.7691**	**0.0002**		**bilat_hippo**	**2.2826**	**0.3025**	**7.5460**	**<0.0001**
	**bilat_thal**	**0.9237**	**0.2108**	**4.3822**	**<0.0001**		bilat_thal	0.2898	0.2544	1.1393	0.2547
	bilat_pfc	0.0273	0.0518	0.5265	0.5986		bilat_pfc	0.0683	0.0616	1.1087	0.2677
	**ICV**	**-0.0042**	**0.0014**	**-3.0229**	**0.0025**		ICV	-0.0016	0.0017	-0.9365	0.3491
	**WMLV**	**0.0877**	**0.0316**	**2.7741**	**0.0056**		WMLV	0.0483	0.0377	1.2803	0.2005
	education	0.0319	0.0665	0.4793	0.6317		education	0.0351	0.0800	0.4390	0.6607
	head motion	-0.0066	0.0222	-0.2990	0.7650		head motion	-0.0238	0.0262	-0.9054	0.3654
	**CEN**	**22.2325**	**5.1428**	**4.3230**	**<0.0001**		CEN	18.3748	6.2798	2.9260	0.0035
	amyloid*CEN	0.0280	0.0566	0.4940	0.6213		**amyloid*CEN**	**0.1632**	**0.0677**	**2.4114**	**0.0160**
Baseline eSPPB	amyloid	-0.0052	0.0020	-2.6326	0.0085	30-month eSPPB	amyloid	-0.0066	0.0024	-2.7818	0.0054
	**age**	0.0201	0.0019	10.5295	**<0.0001**		**age**	**0.0188**	**0.0023**	**8.1985**	**<0.0001**
	gm_vol	0.0001	0.0002	0.4029	0.6870		**gm_vol**	**0.0008**	**0.0003**	**3.1744**	**0.0015**
	wm_vol	-0.0001	0.0003	-0.2156	0.8293		wm_vol	0.0000	0.0003	0.1367	0.8913
	bilat_hippo	0.0086	0.0126	0.6854	0.4931		bilat_hippo	-0.0189	0.0154	-1.2311	0.2184
	bilat_thal	0.0012	0.0105	0.1153	0.9082		bilat_thal	-0.0138	0.0128	-1.0821	0.2793
	bilat_pfc	-0.0043	0.0026	-1.6591	0.0972		**bilat_pfc**	**-0.0075**	**0.0031**	**-2.4041**	**0.0163**
	ICV	0.0000	0.0001	0.3859	0.6996		ICV	-0.0001	0.0001	-1.0022	0.3163
	**WMLV**	**0.0036**	**0.0016**	**2.2834**	**0.0225**		**WMLV**	**0.0071**	**0.0022**	**3.2868**	**0.0010**
	education	0.0025	0.0033	0.7409	0.4588		education	-0.0014	0.0040	-0.3439	0.7310
	head motion	0.0019	0.0011	1.7641	0.0778		head motion	0.0006	0.0013	0.4690	0.6391
	CEN	0.7130	0.2533	2.8147	0.0049		CEN	0.2269	0.3144	0.7216	0.4706
	**amyloid*CEN**	**0.0077**	**0.0028**	**2.7556**	**0.0059**		**amyloid*CEN**	**0.0103**	**0.0034**	**3.0461**	**0.0023**

Significant interactions or main effects in the absence of an interaction are bolded.

CEN - Central Executive Network, SCN - Subcortical Network, DSST - digit symbol substitution test, eSPPB - expanded short physical performance battery

gm_vol - gray matter volume, wm_vol - white matter volume, bilat_hippo - bilateral hippocampal volume, bilat_thal - bilateral thalamus volume

bilat_pfc - bilateral prefrontal cortex volume, ICV - intracranial volume, WMLV - what matter lesion volume, SE- standard error

Table S5. Sensitive analyses using the same 76 participants in all models

Outcome Variable	Independent Variables	Estimate	SE	T score	p-Value	Outcome Variable	Independent Variables	Estimate	SE	T score	p-Value
SCN											
Baseline DSST	amyloid	-0.0424	0.0465	-0.9107	0.3625	30-month DSST	amyloid	-0.1300	0.0565	-2.3005	0.0215
	education	0.0191	0.0673	0.2835	0.7768		education	0.0237	0.0811	0.2920	0.7703
	**age**	**0.1550**	**0.0393**	**3.9438**	**0.0001**		**age**	**0.3027**	**0.0462**	**6.5525**	**<0.0001**
	head motion	0.0098	0.0223	0.4392	0.6605		head motion	-0.0099	0.0263	-0.3766	0.7065
	**SCN**	**15.4126**	**5.2287**	**2.9477**	**0.0032**		SCN	14.9455	6.5211	2.2919	0.0220
	amyloid*SCN	0.0507	0.0606	0.8355	0.4035		**amyloid*SCN**	**0.1708**	**0.0734**	**2.3255**	**0.0201**
Baseline eSPPB	amyloid	-0.0052	0.0023	-2.2749	0.0230	30-month eSPPB	amyloid	-0.0039	0.0028	-1.3885	0.1651
	education	0.0023	0.0033	0.6936	0.4880		education	-0.0018	0.0040	-0.4444	0.6568
	**age**	**0.0207**	**0.0019**	**10.8418**	**<0.0001**		**age**	**0.0196**	**0.0023**	**8.5233**	**<0.0001**
	**head motion**	**0.0027**	**0.0011**	**2.4916**	**0.0128**		head motion	0.0016	0.0013	1.2059	0.2279
	SCN	-0.0014	0.2606	-0.0052	0.9958		SCN	-0.0741	0.3224	-0.2299	0.8182
	**amyloid*SCN**	**0.0071**	**0.0030**	**2.3775**	**0.0175**		amyloid*SCN	0.0058	0.0036	1.6032	0.1090
CEN											
Baseline DSST	amyloid	-0.0427	0.0403	-1.0593	0.2895	30-month DSST	amyloid	-0.0070	0.0024	-2.9211	0.0035
	education	0.0107	0.0671	0.1602	0.8727		education	-0.0016	0.0040	-0.4016	0.6880
	**age**	**0.1533**	**0.0391**	**3.9213**	**0.0001**		**age**	**0.0191**	**0.0023**	**8.3407**	**<0.0001**
	head motion	-0.0032	0.0224	-0.1425	0.8867		head motion	0.0009	0.0013	0.7256	0.4681
	**CEN**	**24.5945**	**5.1880**	**4.7407**	**<0.0001**		CEN	0.3177	0.3136	1.0132	0.3111
	amyloid*CEN	0.0558	0.0572	0.9759	0.3292		**amyloid*CEN**	**0.0108**	**0.0034**	**3.1886**	**0.0014**
Baseline eSPPB	amyloid	-0.0053	0.0020	-2.6961	0.0071	30-month eSPPB	amyloid	-0.1416	0.0483	-2.9289	0.0034
	education	0.0023	0.0033	0.7070	0.4796		education	0.0191	0.0808	0.2369	0.8128
	**age**	**0.0204**	**0.0019**	**10.7515**	**<0.0001**		**age**	**0.3016**	**0.0460**	**6.5582**	**<0.0001**
	head motion	0.0020	0.0011	1.8538	0.0639		head motion	-0.0243	0.0266	-0.9137	0.3609
	CEN	0.7402	0.2521	2.9360	0.0033		CEN	18.9360	6.3346	2.9893	0.0028
	**amyloid*CEN**	**0.0079**	**0.0028**	**2.8253**	**0.0048**		**amyloid*CEN**	**0.2029**	**0.0684**	**2.9650**	**0.0031**

Significant interactions or main effects in the absence of an interaction are bolded.

CEN - Central Executive Network, SCN - Subcortical Network, DSST - digit symbol substitution test, eSPPB - expanded short physical performance battery

SE- standard error
